# Gold leaching by organic base polythionates: new non-toxic and secure technology

**DOI:** 10.1186/2193-1801-3-180

**Published:** 2014-04-07

**Authors:** Vladislav Smolyaninov, Galina Shekhvatova, Mikhail Vainshtein

**Affiliations:** Scientific-industrial firm “Gamma”, Pushchino, 142290 Russia; Institute of Biochemistry and Physiology of Microorganisms RAS, Pushchino, 142290 Russia; Pushchino State Institute of Natural Sciences, Pushchino, 142290 Russia

**Keywords:** Polythionate, Organic base polythionate, Smolyaninov reaction, Gold leaching, Thiosulfate leaching, Cyanide leaching

## Abstract

The article present a review on own experimental and some published data which are related with the gold leaching. It is well-known that the most common and usual process of the leaching with cyanide can be dangerous, needs a great water consumption, and additional costs for remediation of the poisoned and toxic sites. The experimental data described production of poythionates which are not toxic but perspective for the prosperous gold leaching. The paper dedicated to the safe gold leaching with thiosulfates and organic salts of polythionic acids (organic base polythionates). The method of production of these polythionates based on the Smolyaninov reaction is described in stages and in details for the first time. Possible application of the polythionates application in the gold leaching is discussed and its advantages are compared with the gold leaching by cyanation.

## Introduction

Gold leaching is the key stage in process of gold mining. There are a few well-reputed leaching methods using a variety of different reagents. Nowadays the most popular industrial processes include cyanide, thiourea, and thiosulfate leaching methods. The cyanide leaching is the oldest and the leading one (Mudder and Botz 2004; Hilson and Monhemius 2006). However, cyanide is highly toxic and its use leads to chronic poisoning of the soil in the areas of gold mining, causing severe ecological problems.

In comparison to the cyanide leaching, the thiourea leaching is less toxic and more cost-effective, but it is still far from satisfactory too.

Leaching by thiosulfates is more advantageous technology (Muir and Aylmore 2005; Aylmore 2005): it is less expensive and more environment friendly. The low cost of the reagents and no such a big need in the site decontamination make thiosulfate leaching attractive from an economical point of view. The difficulty limiting application of the thiosulfate (S_2_O_3_^2-^) or polythionate (S_x_O_6_^2-^) leaching lies in their industrial production. Use of well-known reactions of polythionates production with halogens may lead i) to increase of the cost and ii) to degradation of equipment due to residual amounts of the halogens.

Till present time, the poythionates leaching is not common. There were a few reasons which limited application of the process. It has been considered for a long time that the compounds of the composition H_2_S_x_O_6_ are rather unstable in aqueous solutions under the form of some salts. For many years it has been considered that the compounds with × = 3-6 known as polythionic acids, are limited by the first four compounds. Polythionates have been neglected for many years due to their instability and to the formation of complex mixtures. Individual polythionate determining in a solution was a complicated task. Nevertheless, polythionates can finds practical application both in general chemistry and in industry.

On the basis of experimental data, we discovered reactions of thiosulfuric acid accompanied with oxidation by mono- and polyatomic sulfur in the presence of “onium”-containing (i.e. ammonium-, sulfonium-, phosphonium-containing) compounds. Thus, it became possible to examine the problem of polythionic acid formation during thiosulfuric acid decomposition (Volynskiy-Smolyaninov reaction) (Smolyaninov and Volynskiy 1965). As well, we succeeded to establish experimentally that the interaction of SO_2_ and of H_2_S in ethanol in the presence of organic bases results in the formation of polythionic acids (here and below – Smolyaninov reaction) (Smolyaninov et al. 2010a).

Unfortunately, the chemical reactions, where structure of the organic base determines the structure of the inorganic acid, are remaining unnoticed in spite of the possibilities they offer to obtain new chemical compounds with very interesting and earlier unknown properties. The following review discusses their possible production and application for innovative hydrometallurgical processes and, as well, as reagents for other new approaches (for analyses of sulfurous springs, industrial and domestic effluents, etc.).

## Formation of organic base polythionates

### Formation of pentathionates in the reaction of acids with thiosulfates in the presence of some organic bases

We observed that acidifying thiosulfate solutions in the presence of some higher amine salts or ammonium base salts results in the precipitation under the form of oil, and the reaction mixture smells hydrogen sulfide (Volynskiy 1959).

The pentathionic acid is formed, after acidifying some thiosulfate in the presence of salts of some amines and quaternary ammonia, according to the equations as follows:123

Hydrogen sulfide emitted under the reaction (2) immediately reacts with the sulfurous gas that had been formed at the thiosulfuric acid decomposition. At this moment, sulfur is emitted again and the whole process repeats from the beginning until complete transformation of thiosulfuric acid to pentathionic acid according to the ultimate Equation (3). The reaction (2) was not described in literature till present.

### Formation of tetra- and pentathionates in reaction of acids with thiosulfates in the presence of organic base salts

During our study of the thiosulfuric acid transformations in the presence of organic bases, we synthesized N-alkylpyridinium, N-alkylquinolinium, and dimethylalkylphenylammonium chlorides containing from 5 to 10 carbon atoms in the alkyl chain (Volynskiy and Smolyaninov 1963). We studied the dependency between the polythionic acid formation and the structure of nitrogen bases in more details, and our investigations showed that in the presence of salts of dimethyldecyl-, dimethylnonyl- or dimethyloctylphenylammonium the thiosulfuric acid is transformed to pentathionic acid according to the Equation (3).

Under the effect of N-alkylpyridinium and N-alkylquinolinium ions, the thiosulfuric acid forms both pentathionic and tetrathionic acids, the last being formed as the main compound in many cases.

At room temperature, the transformation of thiosulfuric acid to tetrathionic or pentathionic acid occurs with rather high yields in the presence of organic base ions, containing an alkyl chain with at least 7-6 carbon atoms. Further reduction of the alkyl chain length leads to a drastic decrease in tetrathionate (and pentathionate) yields and to a respective increase in sulfur yields according to the reaction (1).

Under the effect of a catalyst, namely–an organic base ion, the monatomic sulfur formed during the partial decomposition of the thiosulfuric acid according to the Equation (4) acquires more expressed oxidizing properties, which makes it possible to start a reaction (5) not described in literature. It is easy to understand that the reaction (5) is similar to the reaction of oxidizing thiosulfuric acid with iodine. The hydrogen sulfide emitted reacts with H_2_SO_3_, and sulfur formed in this case reacts again according to the Equation (5). The process is described by the Equation (6):456

It is of interest to clarify the specificity degree of the effect of ammonium ions and if it represents a specific case of a common phenomenon consisting in the effect of the onium ions on the oxidizing capacity of monatomic and diatomic sulfur. We found out that sulfonium and phosphonium ions manifested some activity in the process under study, similar to the ammonium ions activity.

### Formation of organic base polythionates

The research we carried out about the effect of the organic base structure on the preferred formation of tetra- or pentanthionic acid with acidifying the aqueous solution of sodium thiosulfate and the organic base salt enables us to arrive at some general conclusions (Smolyaninov et al. 2012):

1. Primary amines do not favor the formation of polythionic acids.
2. Secondary amines do not favor the formation of polythionic acids, but in the presence of at least one hydrocarbonic radical with rather long chains it is possible to get tetrathionic acid. In the presence of a secondary butyloctadecylamine, we registered the formation of tetrathionic acid.
3. Tertiary amines having one hydrocarbonic radical containing more than 5 carbon atoms favor the formation of tetrathionates, and tertiary amines with 2 or 3 hydrocarbonic radicals of this type form pentathionates, their yields increasing with the increase of the hydrocarbonic chain length.
4. Quaternary ammonium bases, in the presence of two rather long alkyl chains, favor the formation of pentathionates.
5. N-alkylquinolinium, starting from N-hexylquinolinium, favors the preferred formation of tetrathionic acid.
6. N-alkylpyriginium, starting from N-hexylpyridinium, forms a mixture of polythionates.
7. Dimethylalkylphenylammonium, starting from dimethylhexylphenylammonium, forms pentathionates.
8. N-methyl-8-alcoxyquinolinium and 8-alcoxyquinolinium with an alkyl radical containing 5 and more carbon atoms form tetrathionates.
9. Diisoamyl-β-acyloxyethylammonium ions and diisoamyl-β-alkyloxyethylammonium ions form pentathionates.
10. Sulfonium ions, depending on the structure, form tetra- and pentathionates.

In all the cases, only neutral salts of polythionic acids were formed. So, a reaction of forming organic base polythionates with acidification of thiosulfate in the presence of organic base salts was discovered:7

where R_1_R_2_R_3_ are hydrocarbonic radicals of С_1_ to С_18_, one of them being at least С_6_,

N is sulfonium, phosphonium or heteroatomic nitrogen,

Hal is Cl^-^, Br^-^, J^-^, NO_3_^-^ or other anions,

n is the number of sulfur atoms of 3 to 6, mainly 4-5.

Despite the fact that this presentation is a review, this equation is given for the first time in scientific literature. This Volynskiy-Smolyaninov reaction was published only as a “Method of obtaining polythionates of higher amines and onium compounds” claimed with the USSR Author’s certificate (some kind of the former Soviet patent) (Smolyaninov and Volynskiy 1965).

To obtain the organic base polythionates, thiosulfuric acid or sulfurous acid is used in excess which leads to the formation of acid salts of these acids.

Oxidation of the forming salts of mono-, di-, triatomic and polyatomic sulfur leads to the formation of trithionates, tetrathionates, pentathionates, etc.

The presence of an organic base favors the increase in the oxidative capacity of sulfur, and the number of hydrocarbonic chains and their length determine the accessibility of the hydrogen from the acid for the oxidizer and the stability of the chain to be formed. Assuming that the alkyl radicals protect the sulfur chain against hydrolysis, one can understand why 2 or 3 long alkyl radicals in tertiary amines or in quaternary ammonium bases shift the reaction to the formation of longer polythionic acids, and in the presence of OH^-^ groups in alkyl chains, the polythionates are not formed, the lower amines neither favoring the formation of polythionates.

The length of a chain with 5 C-atoms is almost equal to the chain of 4 S-atoms, and this fact seems to determine the minimum alkyl chain lengths in bases, starting from which it becomes possible to form polythionic acids while acidifying thiosulfate in the presence of organic bases.

The excess of HSO_3_^-^ or HS_2_O_3_^-^ ions and the structure of the organic base finally determine the preferred yield of such or another polythionic acid.

These experimental data showed for the first time that the length of the S_n_ chain in organic base polythionates depends on the length of radicals and their amount (Smolyaninov 1970).

In general, approximately 140 new chemical compounds were synthesized when studying the reactions of the organic base polythionate formation with acidifying the thiosulfate and the organic base salt in water and those of the organic base polythionate formation at the interaction of an organic base with SO_2_ and H_2_S in alcohol.

Interaction of alcohol solutions of SO_2_ and H_2_S (in ethanol) led to quantitative formation of sulfur. When organic bases are present in the reaction mixture, then there are formed polythionates of these organic bases with a very high yield and some amount of sulfur as a result of the excess of SO_2_ and H_2_S. The preferred yield and relation of various polythionates depended on the structure of the organic base (7) (Smolyaninov et al. 2010a).8

This novel chemical reaction, the Smolyaninov reaction, was patented as a “Method of obtaining organic base polythionates” (RU Patent No 2404948) (Smolyaninov et al. 2010a).

## Polythionates in the gold leaching

It was proposed to use polythionate organic bases as efficient gold eluting agents from an anion exchanger, after leaching with a thiosulfate (Fleming et al. 2002; Matthew 2007). Thiosulfate leaching of gold represents a potentially attractive alternative to a respective cyanation process at least for three types of auriferous ores. Firstly, in auriferous ores containing an organic carbon material, the recovery of gold by thiosulfate leaching is generally significantly higher due to the insensibility of the thiosulfate complex of gold to the preg-robbing or, in other words, to the re-sorption or solution depletion. In the second place, Au, Cu-containing ores often are not convenient for the cyanation process due to high cyanide consumption by copper in the ore, which leads to an unacceptably high cost. Thiosulfate does not easily react with copper minerals, and the lower reagent cost and the lower sulfate consumption compared to cyanides lead to a lower cost in such a situation. Finally, there are some layers of auriferous ore that cannot be treated by cyanation for being located in a delicate environment. The thiosulfate leaching reduces the load onto environment since the chemical reagents used in this process are already used in agriculture.

As shown in the literature (Smolyaninov et al. 2012), the gold-thiosulfate complex is eluted by polithionates that are produced with the use of iodine, bromine, and hydrogen peroxide as oxidants. Thus minor residual amounts of iodine and bromine lead to very severe corrosion of the process equipment. To avoid adverse effects, we proposed to apply organic base polythionates for eluting a gold-containing complex from a sorbing agent (Smolyaninov et al. 2010a, b).

Method of gold recovery from sulfide auriferous ores has already been proposed (Smolyaninov et al. 2010b). The invention relates to the method of gold recovery from powdered sulfide auriferous ores after their unlocking by bacterial leaching or by oxidation roasting, or by autoclave oxidation. Examples of bioleaching are bacterial oxidation of insoluble metal sulfides with production of sulfuric acid on spot. It is interesting that some leaching bacteria can produce also other sulfur compounds including the mentioned polythionates (Vatsurina et al. 2005). Low concentration of metal sulfides in ores makes no problem for bacteria which ignore surrounding mineral dumps. Most number of modern bioleaching technologies is oriented to application of super-acidophilic bacteria, which produce sulphuric acid and can live at pH lower than 1.0. Toxicity of the leached heavy metals is not a limiting factor because some bacterial strains can be adopted (Astudillo and Acevedoa 2008) or modified with the specific plasmid of resistance (Filonov et al. 2009).

The proposed method of the gold recovery comprises leaching with a solution of a mixture of sodium and ammonium hydrosulfite and thiosulfate, by sorption of the thiosulfate-gold complex on a highly basic anion exchanger and by isolation of the highly basic anion exchanger, the sorption being carried out 2-10 hours after the leaching. Then the thiosulfate-gold complex is eluted with a solution of organic and inorganic base polythionates with concentration of 0.2 to 10%. Gold is isolated from the gold-containing eluate by a decomposition reaction or by an electrochemical method. In this case, the decomposition reaction is performed with such metals as magnesium, zinc or iron, or with sulfides. Then the highly basic anion exchanger is regenerated with a solution of a mixture of sodium and ammonium sulfite and sulfate. After the highly basic anion exchanger regeneration, it is transferred to the process of the thiosulfate-gold complex sorption. The technical advantage resides in a faster leaching, a higher gold yield and a lower consumption of the leaching agent.

Method of obtaining organic base polythionates was not discussed in international scientific literature earlier but just claimed with a patent (Smolyaninov et al. 2010a). Meanwhile, the invention relates to a method of obtaining organic base polythionates used for biological hydrometallurgy processes (Smolyaninov et al. 2010b). It is impossible to obtain polythionates well soluble in water, by known processes, since for a successful process run, it is necessary for the organic base to have at least one aliphatic radical with the carbon atom number of at least 7. It is propose a method of obtaining organic base polythionates, in which the organic base solution in an organic solvent is treated with sulfurous gas and later by hydrogen sulfide. After separating precipitated sulfur and distilling off the organic solvent, the organic base polythionate is recovered. No limitations exist for the organic base structure in this reaction (9).

Summarizing the general formula of the Smolyaninov reaction, the reaction to form any organic bases (Smolyaninov et al. 2010a), it can be presented as follows:9

where R_1_R_2_R_3_ are hydrocarbon radicals, С_1_ –С_18_ and N represents both ammonium and other onium bases.

In a particular case, while using triethylaimines10

where the reaction gives colorless oily liquid very similar to glycerol.

Since the structure of the polythionic acid depends on the base structure, the number of sulfur atoms can be different, i.e. 3, 5, 6, etc. These equations are presented for the first time.

## Conclusions

The different stages, conditions, and schemes of the polythionates production and their application for gold leaching are described. In whole, an innovative approach is shown suggesting that the use of polythionates or of combined thiosulfate leaching together with elution of gold complexes with organic bases polythionates makes the gold leaching process in mining more safe and thus more economical than cyanation.

It is the first case when the Smolyaninov reaction to produce polythionates is presented in details with the and affiliated details.

The bench-scale experimental data suggest some future scaling up of the process and, as well, the possible polythionates application in fields of the gold mining and the recovery of valuable metals from solid wastes.
